# Synthetic lethal short hairpin RNA screening reveals that ring finger protein 183 confers resistance to trametinib in colorectal cancer cells

**DOI:** 10.1186/s40880-017-0228-1

**Published:** 2017-07-31

**Authors:** Rong Geng, Xin Tan, Zhixiang Zuo, Jiangxue Wu, Zhizhong Pan, Wei Shi, Ranyi Liu, Chen Yao, Gaoyuan Wang, Jiaxin Lin, Lin Qiu, Wenlin Huang, Shuai Chen

**Affiliations:** 1 State Key Laboratory of Oncology in South China, Collaborative Innovation Center of Cancer Medicine, Sun Yat-sen University Cancer Center, 651 Dongfeng Road East, Guangzhou, 510507 Guangdong P. R. China; 2 Department of Experimental Research, Sun Yat-sen University Cancer Center, Guangzhou, 510060 Guangdong P. R. China; 3 Department of Colorectal Surgery, Sun Yat-sen University Cancer Center, Guangzhou, 510060 Guangdong P. R. China; 4 Guangdong Esophageal Cancer Institute, Sun Yat-sen University Cancer Center, Guangzhou, 510060 Guangdong P. R. China; 5 Guangdong Provincial Key Laboratory of Tumor-Targeted Drugs and Guangzhou Enterprise Key Laboratory of Gene Medicine, Guangzhou Doublle Bioproducts Co. Ltd., Guangzhou, 510507 Guangdong P. R. China

**Keywords:** Synthetic lethal, Colorectal cancer, Ring finger protein 183, Mitogen-activated extracellular signal-regulated kinase 1/2, Trametinib

## Abstract

**Background:**

The mitogen-activated extracellular signal-regulated kinase 1/2 (MEK1/2) inhibitor trametinib has shown promising therapeutic effects on melanoma, but its efficacy on colorectal cancer (CRC) is limited. Synthetic lethality arises with a combination of two or more separate gene mutations that causes cell death, whereas individual mutations keep cells alive. This study aimed to identify the genes responsible for resistance to trametinib in CRC cells, using a synthetic lethal short hairpin RNA (shRNA) screening approach.

**Methods:**

We infected HT29 cells with a pooled lentiviral shRNA library and applied next-generation sequencing to identify shRNAs with reduced abundance after 8-day treatment of 20 nmol/L trametinib. HCT116 and HT29 cells were used in validation studies. Stable ring finger protein 183 (RNF183)-overexpressing cell lines were generated by pcDNA4-myc/his-RNF183 transfection. Stable RNF183-knockdown cell lines were generated by infection of lentiviruses that express RNF183 shRNA, and small interference RNA (siRNA) was used to knock down RNF183 transiently. Quantitative real-time PCR was used to determine the mRNA expression. Western blotting, immunohistochemical analysis, and enzyme-linked immunosorbent assay (ELISA) were used to evaluate the protein abundance. MTT assay, colony formation assay, and subcutaneous xenograft tumor growth model were used to evaluate cell proliferation.

**Results:**

In the primary screening, we found that the abundance of RNF183 shRNA was markedly reduced after treatment with trametinib. Trametinib induced the expression of RNF183, which conferred resistance to drug-induced cell growth repression and apoptotic and non-apoptotic cell deaths. Moreover, interleukin-8 (IL-8) was a downstream gene of RNF183 and was required for the function of RNF183 in facilitating cell growth. Additionally, elevated RNF183 expression partly reduced the inhibitory effect of trametinib on IL-8 expression. Finally, xenograft tumor model showed the synergism of RNF183 knockdown and trametinib in repressing the growth of CRC cells in vivo.

**Conclusion:**

The RNF183-IL-8 axis is responsible for the resistance of CRC cells to the MEK1/2 inhibitor trametinib and may serve as a candidate target for combined therapy for CRC.

**Electronic supplementary material:**

The online version of this article (doi:10.1186/s40880-017-0228-1) contains supplementary material, which is available to authorized users.

## Background

Colorectal cancer (CRC) is the third most common malignancy worldwide [1]. The mechanisms underlying the initiation and development of CRC are complex and involve many key pathways, such as the Wnt, mitogen-activated protein kinase (MAPK), and transforming growth factor-β (TGF-β) signaling pathways [2–5]. Mitogen-activated extracellular signal-regulated kinase 1/2 (MEK1/2) is a dual threonine- and tyrosine-recognizing kinase that phosphorylates and activates kinases in the MAPK signaling pathway [6]. Although MEK inhibitors are currently used in targeted therapy for melanoma, MEK inhibitor monotherapy (such as trametinib) was found to be ineffective for CRC [6]. Many mechanisms of resistance to trametinib have been identified, including activation of upstream receptor tyrosine kinases and stimulation of parallel signals bypassing MEK inhibition to reactivate extracellular signal-regulated kinase (ERK) signaling [7]. Therefore, rational drug combinations, potentially with agents targeting proteins that confer resistance to MEK inhibitors, are likely to improve the therapeutic efficacy.

Synthetic lethality was originally identified in gene function studies in drosophila, which means that a combination of two or more separate gene mutations causes cell death, whereas individual mutations keep cells alive [8]. This strategy could be used to identify therapeutic targets against cancer. For example, ovarian cancer cells with mutations of the breast cancer susceptibility genes 1 and 2 (*BRCA1* and *BRCA2*) have good response to poly(ADP-ribose) polymerase (PARP) inhibitors [8]. The use of genome-wide approaches, such as short hairpin RNA (shRNA) and clustered regularly interspaced short palindromic repeats (CRISPR)-based screening libraries, plus next-generation sequencing largely facilitate the process of identifying genes that confer synthetic lethality with drugs. An increasing number of targets have been identified, and new combination strategies were developed and validated in preclinical studies [9, 10].

The RING finger (RNF) protein family is a complex set of proteins containing a RING finger domain with 40–60 amino acids [11, 12]. Although it has been reported that many RNF members play key roles in carcinogenesis [13–15], their association with CRC is largely unknown. The expression of RNF183 was found to be up-regulated in inflammatory bowel disease (a causative factor for CRC) and to activate the classical nuclear factor-kappaB (NF-κB) pathway [16–18]. It has not, however, been reported whether RNF183 plays a role in CRC. In this study, we used a synthetic lethal shRNA screening to identify genes conferring resistance to trametinib in CRC cell line HT29. Furthermore, we investigated whether RNF183 could serve as a possible target for combined therapy for CRC.

## Methods

### Cell lines and cell culture

Colorectal cancer cell lines HCT116 and HT29 were purchased from the American Type Culture Collection (ATCC, Rockville, MD, USA) and cultured in McCoy’s 5A medium (Gibco, Grand Island, NY, USA) supplied with 10% fetal bovine serum (Hyclone, Irvine, CA, USA) in a humidified atmosphere of 5% CO_2_ at 37 °C. Thawed cells from liquid nitrogen were used within the first three passages.

### Plasmids, antibodies, and reagents

The plasmid pcDNA4-myc/his was purchased from Invitrogen (Carlsbad, CA, USA). RNF183-expressing plasmid pcDNA4-myc/his-RNF183 was constructed by Genewiz (Suzhou, Jiangsu, China). Lentiviral vector LV2 was purchased from GenePharma (Shanghai, China), and the sequences of negative control shRNA (shNC) and *RNF183* shRNA (shRNF183) were inserted into this vector, respectively. The sequence of *RNF183* that were targeted by shRNA as well as sequences of *RNF183* and interleukin-8 (*IL*-*8*) that were targeted by small interfering RNA (siRNA) (RiboBio Company, Beijing, China) are listed in Table 1.

**Table 1:** Sequences of human ring finger protein 183 (RNF183) and interleukin-8 (IL-8) that were targeted by small interfering RNAs (siRNAs) and short hairpin RNA (shRNA)

Name	Targeted sequence
siRNF183	5′-CCACCAUGUCAUCCUGGAA-3′
siRNF183-2	5′-GCAUCUUUGCCUACCUGAU-3′
shRNF183	5′-CCACCAUGUCAUCCUGGAA-3′
siIL-8	5′-GCCAAGGAGUGCUAAAGAA-3′

The antibodies used are as follows: anti-human RNF183 antibody (dilution of 1:1000 for Western blotting, 1:200 for immunohistochemical analysis [IHC], ab197321; Abcam, Cambridge, UK), anti-β-actin antibody (dilution of 1:200 for Western blotting, sc-47778; Santa Cruz Biotechnology, Santa Cruz, CA, USA), anti-mouse IgG HRP conjugate (dilution of 1:8000 for Western blotting, W402B; Promega, Madison, WI, USA), anti-rabbit IgG HRP conjugate (dilution of 1:2000 for Western blotting, #7074P2; Cell Signaling Technology, Wellesley, MA, USA), and anti-Ki-67 antibody (dilution of 1:200 for IHC, #12202; Cell Signaling Technology).

The protein level of IL-8 in culture supernatant was determined using a human IL-8 enzyme-linked immunosorbent assay (ELISA) kit (Proteintech, Wuhan, Hubei, China). Trametinib was obtained from Selleck Chemicals (Houston, TX, USA) and dissolved in dimethyl sulfoxide (DMSO) (Sigma-Aldrich, St. Louis, MO, USA) at 50 mmol/L and stored at −80 °C for in vitro experiments. It was reconstituted in distilled H_2_O containing 0.5% hydroxypropyl methylcellulose (Sigma-Aldrich) and 0.2% Tween-80 (Sigma-Aldrich) for in vivo experiments. The reagent 3-(4,5-dimethyl-thiazol-2-yl)-2,5-diphenyltetrazolium bromide (MTT) was also obtained from Sigma-Aldrich.

### shRNA screening and bioinformatics analysis

A lentiviral shRNA library (catalog number: SHPH01) consisting of over 150,000 shRNA constructs targeting more than 15,000 human genes was obtained from Sigma-Aldrich. HT29 cells were transfected with the lentiviral shRNA library at a multiplicity of infection of 0.4, and stable cell lines were generated by puromycin selection for 7 days. Then, these stable cells were divided into two groups and treated with DMSO or 20 nmol/L trametinib for an additional 8 days. The genomic DNA of treated stable cells was extracted, and the shRNA fragment was amplified using primers flank the insertion site of the lentiviral vector, which were provided by the library manufacturer. The amplicon was subjected to next-generation sequencing using a Hiseq 4000 sequencer (Illumina, San Diego, CA, USA), and the abundance of each shRNA in each treatment group was calculated. Genes were considered responsible for trametinib resistance when the following three conditions were met: (1) the abundance of the shRNA for this gene in the DMSO group was higher than 25 per million sequences; (2) the abundance of the shRNA for this gene in the trametinib group was lower than that in the DMSO group; and (3) the *P* value for the difference in the shRNA abundance between the DMSO and trametinib groups was smaller than 0.01.

### RNA extraction and quantitative real-time PCR

Total RNA from HCT116 and HT29 cells was extracted using Trizol (Invitrogen). RNA was reversely transcribed to synthesize complementary DNA (cDNA) using reverse transcription-related reagents (Promega). SYBR^®^ Green was purchased from Bio-Rad (Hercules, CA, USA) for quantitative real-time PCR (qPCR) [19–21]. The primer sequences of target genes, including *RNF183*, selectin E (*SELE*), intercellular adhesion molecule 1 (*ICAM1*), matrix metallopeptidase 9 (*MMP9*), plasminogen activator urokinase (*PLAU*), C-X-C motif chemokine receptor 4 (*CXCR4*), *IL*-*8*, interleukin-6 (*IL*-*6*), and glyceraldehyde-3-phosphate dehydrogenase (*GAPDH*) are listed in Table 2. *GAPDH* was used as an endogenous control. Gene expression levels in each cDNA sample were normalized to the internal *GAPDH* levels, and the values were further normalized to control conditions (e.g., cells transfected with pcDNA4-myc/his, cells transfected with non-specific control, and cells without trametinib treatment).

**Table 2:** Primer sequences for quantitative real-time PCR

Gene	Forward sequence	Reverse sequence	Product length (bp)
*RNF183*	5′-CGAAAAGCTTGAAGGACTGG-3′	5′-TGAAGCAGCTCCAGTGAGAA-3′	166
*SELE*	5′-GGACACAGCAAATCCCAGTT-3′	5′-CTCCAATAGGGGAATGAGCA-3′	266
*ICAM1*	5′-GAGATCACCATGGAGCCAAT-3′	5′-CTGACAAGTTGTGGGGGAGT-3′	120
*MMP9*	5′-TTGACAGCGACAAGAAGTGG-3′	5′-CCCTCAGTGAAGCGGTACAT-3′	129
*PLAU*	5′-GCCATCCCGGACTATACAGA-3′	5′-ACACAGCATTTTGGTGGTGA-3′	195
*CXCR4*	5′-TGAGAAGCATGACGGACAAG-3′	5′-GACGCCAACATAGACCACCT-3′	156
*IL*-*8*	5′-CTGCGCCAACACAGAAATTAT-3′	5′-CATCTGGCAACCCTACAACAG-3′	214
*IL*-*6*	5′-TCAATGAGGAGACTTGCCTGGTGA-3′	5′-TCATCTGCACAGCTCTGGCTTGTT-3′	120
*GAPDH*	5′-CTCCTCCTGTTCGACAGTCAGC-3′	5′-CCCAATACGACCAAATCCGTT-3′	185

*RNF183* ring finger protein 183, *SELE* selectin E, *ICAM1* intercellular adhesion molecule 1, *MMP9* matrix metallo peptidase 9, *PLAU* plasminogen activator urokinase, *CXCR4* C-X-C motif chemokine receptor 4, *IL*-*8* interleukin-8, *IL*-*6* interleukin-6, *GAPDH* glyceraldehyde-3-phosphate dehydrogenase

### Western blotting

After 48-h treatment with trametinib, HT29 and HCT116 cells were lysed with cell lysis buffer containing phosphatase inhibitor and protease inhibitor (Sigma-Aldrich). The supernatants were collected and measured using a BCA protein assay kit (KeyGEN BioTECH, Nanjing, Jiangsu, China). For each sample, 35 μg of protein lysis was used for immunoblotting. Proteins were separated by 10% sodium dodecyl sulfate–polyacrylamide gel electrophoresis and transferred to a polyvinylidene difluoride membrane. The protein band was visualized using electrochemiluminescence (KeyGEN BioTECH).

### Establishment of stable cell lines

HT29 and HCT116 cells were transfected with pcDNA4-myc/his-RNF183 or pcDNA4-myc/his using Lipofectamine2000 (Invitrogen). After 48-h transfection, the cells were treated with 300 μg/mL zeocin (Invitrogen) for an additional 12 days to select cells stably expressing RNF183 and control cells. The shRNF183 and shNC lentiviral vectors were generated by GenePharma and transfected into HCT116 and HT29 cells. Stable cell lines with RNF183 knockdown and control cells were selected in the presence of 3 μg/mL puromycin for 7 days, respectively.

### Cell viability, apoptosis, and cytotoxicity assays

Cell viability was measured with MTT assay. Briefly, cells were planted in a 96-well plate (3000 cells per well) and incubated for 1, 2, and 3 days with or without trametinib (20 nmol/L). The medium was then replaced with 5 mg/mL MTT solution, followed by incubation for another 4 h. Subsequently, MTT solution was removed, and DMSO was added to dissolve crystals. Finally, the absorbance was measured at 490 nm (*A*_490_). Each measurement was performed in triplicate. The proliferation rate was calculated: relative proliferation rate = *A*_490_ of the siIL-8 group/*A*_490_ of the siNC group × 100%.

Annexin V-fluorescein isothiocyanate (FITC)/propidium iodide (PI) apoptosis assay kit was purchased from BestBio (Nanjing, Jiangsu, China), and lactate dehydrogenase (LDH) secretion kit was purchased from Promega. Cells were seeded in 6-well plates at the density of 3 × 10^5^ cells per well and treated with trametinib for 72 h. The supernatant was collected for cytotoxicity evaluation, i.e., LDH detection. Briefly, 50 μL supernatant and 50 μL CytoTox 96^®^ reagent (Promega) were added into each well of a 96-well plate. After 30-min incubation at room temperature, 50 μL of stop solution was added to each well. Finally, the absorbance, *A*_490_, was measured. The LDH release rate was calculated using the following formula: LDH release rate = *A*_490_ of the experimental LDH release group/*A*_490_ of the reference maximum LDH release group × 100%.

The adherent cells were prepared for apoptosis assay. Briefly, cells were trypsinized and washed twice with 1× phosphate buffer saline (PBS) and re-suspended in binding buffer. Next, 5 μL annexin V-FITC and 10 μL PI were transferred into cell suspensions. After incubation for 15 min at 4 °C, apoptotic cells were detected on FACSCalibur (BD, Heidelberg, Germany).

### Colony formation assay

For colony formation assay, stable RNF183-overexpressing and control cells transfected with pcDNA4-myc/his were planted in 6-well flat-bottom plates (3 × 10^5^ cells per well). When the cells were 50%–60% confluent, siRNA targeting IL-8 or control siRNA (Ribobio) were transiently transfected into cells using Lipofectamine2000. After 48 h, cells were digested with trypsin (Gibco) and planted into new 6-well plates at a density of 500 cells per well. After being cultured in an incubator for 10 days, the medium was discarded, and cells were washed twice with 1× PBS. Then, the colonies were stained with 5% crystal violet for 15 min, and visible colonies containing at least 50 cells were counted.

### Xenograft tumor growth model

All animal experiments strictly complied with institutional ethical and safety guidelines (Institutional Animal Welfare and Ethics Committee, Sun Yat-sen University Cancer Center, Guangzhou, Guangdong, China). 5- to 6-week-old female BALB/c nude mice were purchased from SLAC Laboratory Animal Inc. (Shanghai, China). We selected HCT116 cells for in vivo experiments. Twenty-four mice were equally distributed into two groups. They were inoculated by subcutaneous injection of 1 × 10^6^ stable shRNF183- or shNC-expressing HCT116 cells. After 7 days, when the median tumor volume exceeded 100 mm^3^, each group was again divided into a trametinib treatment subgroup and a vehicle injection subgroup (each subgroup contained 6 mice), with daily oral administration of 300 μg/kg trametinib or vehicle (distilled H_2_O containing 0.5% hydroxypropyl methylcellulose and 0.2% Tween-80) for 23 days. Tumor length and width were measured every 4 days until the completion of trametinib treatment. The volume (*V*) was calculated using the conventional formula: *V* = (*L* × *W* ^2^)/2, where “*L*” and “*W*” are the length and width of the tumor. Finally, the mice were euthanized, and tumors were resected and photographed. Tumor size and weights were measured. Total RNA was extracted from tumors for IL-8 detection as described above. The remaining tumor specimens were fixed in 4% formaldehyde and then embedded in paraffin to make slices for Ki-67, RNF183, and hematoxylin–eosin (H&E) staining.

### Immunohistochemical (IHC) assay

IHC assay was conducted using the DAB Detection Kit (GeneTech, Shanghai, China). Slides were immersed into 0.01 mol/L citrate buffer (ZSGB-BIO, Beijing, China) and boiled at 98 °C for 15 min in a microwave for antigen retrieval. Endogenous peroxidases were blocked with 3% H_2_O_2_ in methanol for 30 min, and non-specific protein binding was blocked with staining blockers (GeneTech). The samples were incubated with anti-RNF183 antibody or anti-Ki-67 antibody at 4 °C overnight. Then, the slides were washed three times with 1× PBS and incubated with the secondary antibody (GeneTech) at room temperature for 1 h. Then, DAB solution was added to slides for staining according to the manufacturer’s protocol. The tissues on slides were fixed and photographed using a microscope camera (Nikon, Tokyo, Japan).

### Statistical analysis

Statistical analyses were performed using SPSS version 16.0 software (SPSS Inc., Chicago, IL, USA). Student’s t test was used for comparisons. *P* values less than 0.05 were considered statistically significant.

## Results

### Synthetic lethal shRNA screening identified RNF183 as a trametinib-resistant gene

**Fig. 1:**
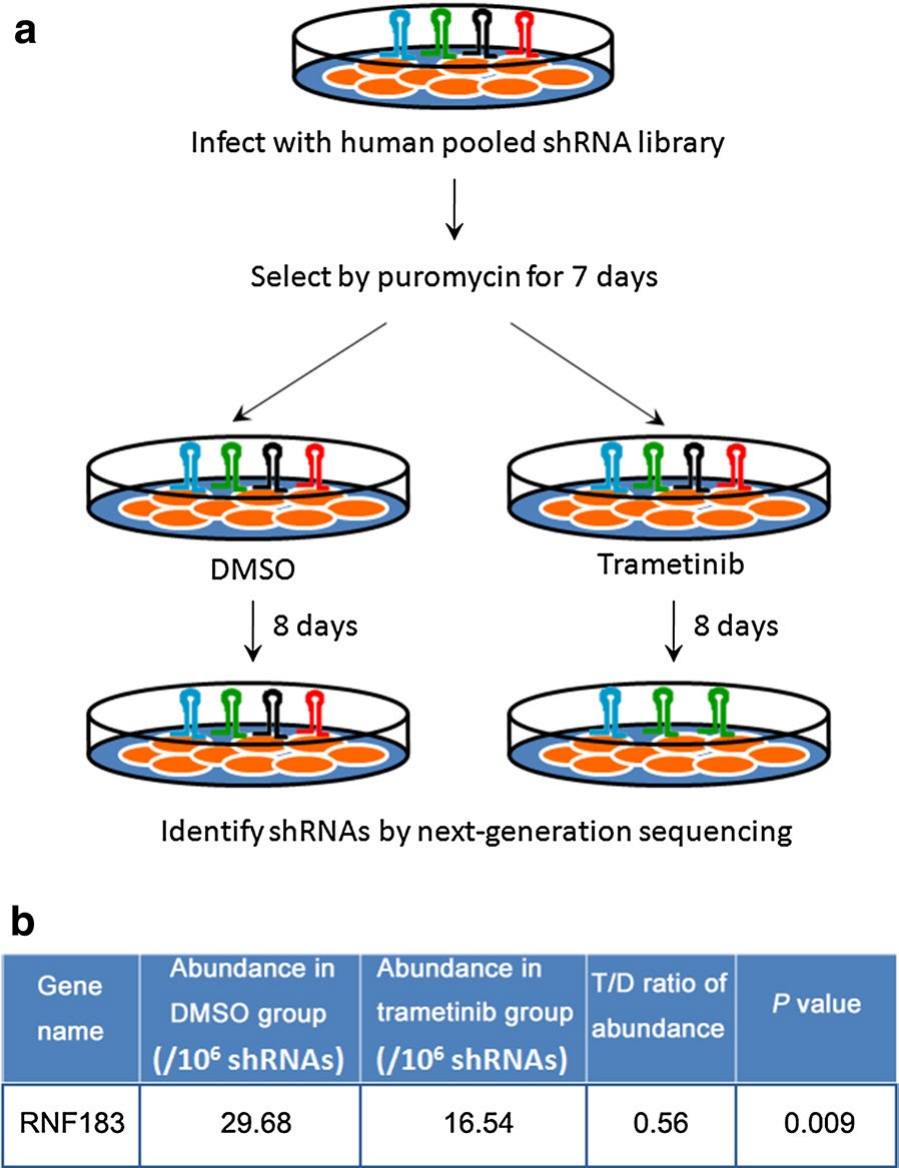
Synthetic lethal short hairpin RNA (shRNA) screening shows that ring finger protein 183 (RNF183) confers resistance to trametinib treatment in colorectal cancer (CRC) cells. **a** HT29 cells were infected with a pooled shRNA library, and the puromycin-selected stable cells were treated with dimethyl sulfoxide (DMSO) or 20 nmol/L trametinib for 8 days. Then, shRNA sequences were amplified by PCR, and next-generation sequencing was conducted to calculate their abundance. **b** The abundance of RNF183 shRNA decreased in cells treated with trametinib compared with those treated with DMSO. T/D ratio of abundance, the ratio of abundance in the trametinib group to abundance in the DMSO group

To identify genes conferring resistance to trametinib, we infected HT29 cells with a lentiviral shRNA library and treated the cells with DMSO or trametinib for 8 days. Subsequently, the shRNA insertions in genomic DNA were amplified and sequenced to calculate their abundance in DMSO or trametinib group (Fig. 1a). A total of 898 shRNAs representing 808 genes were identified by the screening (Additional file 1: Table S1). Several genes have been previously reported to confer resistance to trametinib, such as cyclin-dependent kinase 6 (*CDK6*) [22], histone deacetylase (*HDAC*) [23], and fibroblast growth factor receptor (*FGFR*) [24]. Since the abundance of *RNF183*-targeted shRNA was significantly decreased in the trametinib group (*P* < 0.05) (Fig. 1b), and since it has been reported to play an important role in inflammatory bowel disease [16], which is a causative factor of CRC, we selected this gene for further experiments.

### RNF183 expression was increased after trametinib treatment and conferred drug resistance in CRC cells

**Fig. 2:**
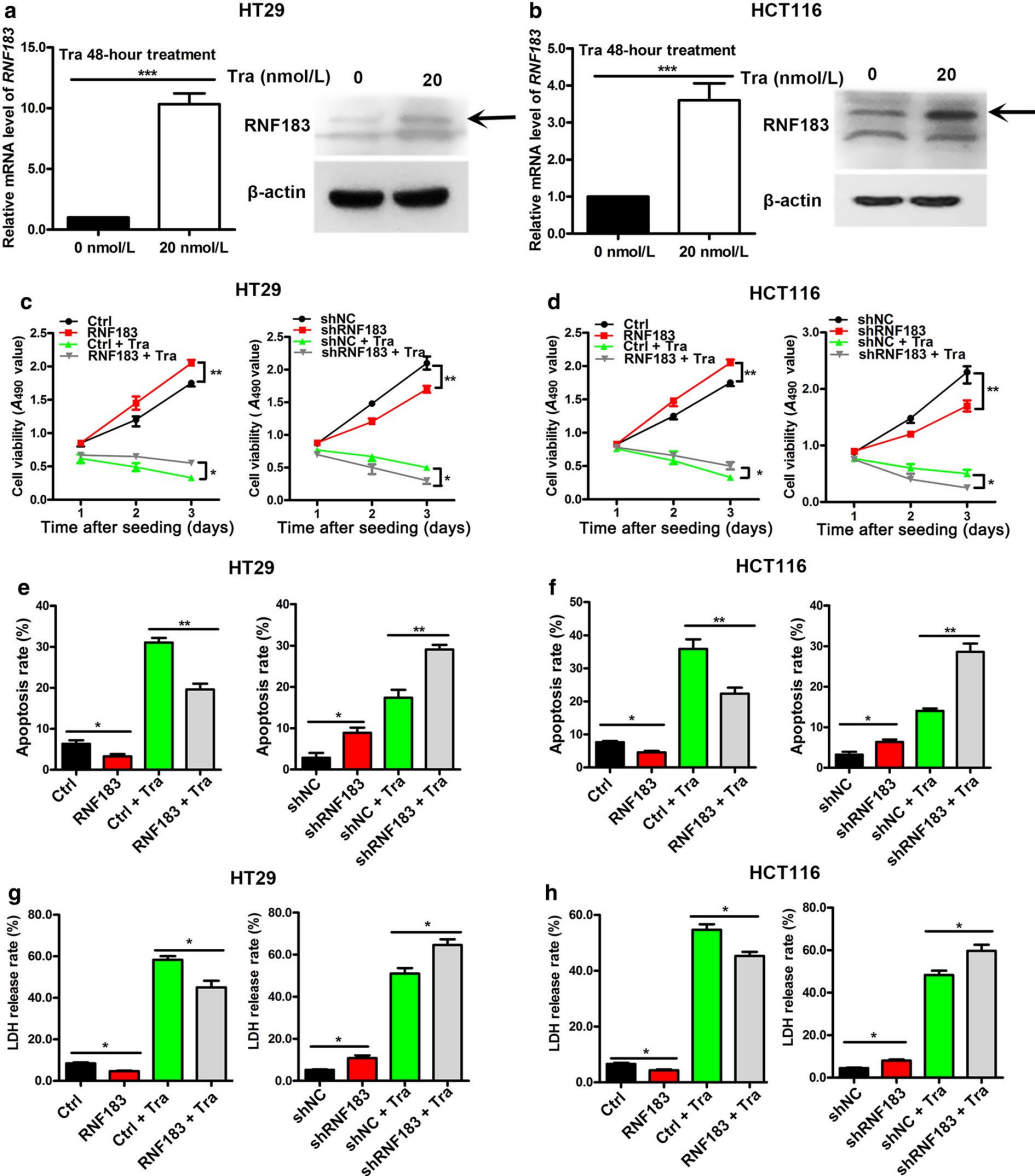
RNF183 expression was increased after trametinib treatment, and its knockdown synergistically repressed the proliferation of CRC cells in combination with trametinib. *RNF183* mRNA abundance was examined using quantitative real-time PCR (qPCR), and its protein level was detected using Western blotting in HT29 (**a**) and HCT116 cells (**b**) with or without 20 nmol/L trametinib for 48 h. *Arrows* indicate the band of RNF183, which was obviously induced by trametinib. Effects of trametinib treatment in combination with RNF183 overexpression or knockdown on the proliferation of HT29 (**c**) and HCT116 cells (**d**) measured using MTT assay. RNF183 conferred resistance to trametinib-induced inhibition on proliferation in both cell lines. Effects of trametinib treatment in combination with RNF183 overexpression or knockdown on the apoptosis of HT29 (**e**) and HCT116 cells (**f**). Effects of trametinib treatment in combination with RNF183 overexpression or knockdown on lactate dehydrogenase (LDH) release of HT29 (**g**) and HCT116 cells (**h**). Experiments were repeated three times. Data are presented as mean ± standard deviation (SD). *P < 0.05, **P < 0.01, ***P < 0.001. *Tra* trametinib, *Ctrl* stable cell lines transfected with pcDNA4-myc/his, *RNF183* stable cell lines transfected with pcDNA4-myc/his-RNF183, *shNC* stable cell lines transfected with lentiviral vectors of negative control shRNA, *shRNF183* stable cell lines transfected with lentiviral vectors of RNF183-targeted shRNA

We first evaluated the expression of RNF183 after trametinib treatment. In HT29 (Fig. 2a) and HCT116 cells (Fig. 2b), both mRNA and protein levels of RNF183 were significantly increased after a 48-h treatment of trametinib. To evaluate the function of RNF183 in trametinib resistance, we generated stable HT29 and HCT116 cells with RNF183 overexpression or knockdown. Results from the MTT assay showed that RNF183 overexpression largely increased the resistance of HT29 cells to trametinib-induced inhibition on proliferation (Fig. 2c, left), and RNF183 knockdown diminished the proliferation of cells in the same condition (Fig. 2c, right). Experiments in HCT116 cells showed similar results (Fig. 2d) (all *P* < 0.05).

Next, we evaluated the effects of RNF183 on apoptotic and non-apoptotic death of CRC cells. RNF183 overexpression significantly decreased the percentages of apoptotic cells in both HT29 and HCT116 cells (all *P* < 0.05) (Fig. 2e, f, left), whereas RNF183 knockdown promoted apoptosis (all *P* < 0.05) (Fig. 2e, f, right) with or without trametinib treatment. LDH release is a biomarker for the occurrence of cytotoxicity and cytolysis that could reflect non-apoptotic death. We found that RNF183 overexpression markedly inhibited trametinib-induced LDH release from HCT116 and HT29 cells (all *P* < 0.05) (Fig. 2g, h, left), whereas RNF183 knockdown increased LDH release in both cell lines with or without trametinib treatment (all *P* < 0.05) (Fig. 2g, h, right).

### IL-8 expression was induced by RNF183 to promote cell growth

**Fig. 3:**
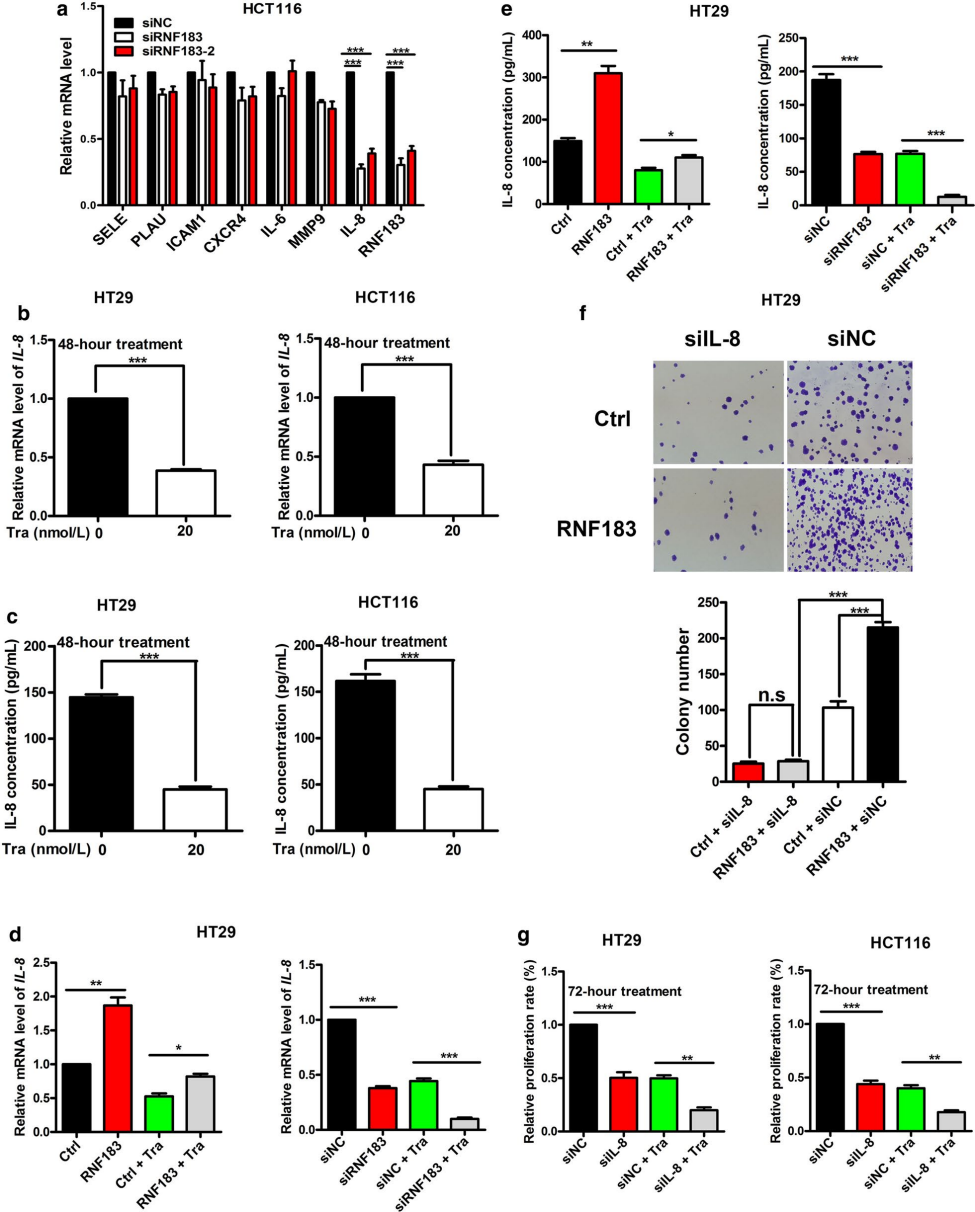
Induction of interleukin-8 (*IL*-*8*) transcription by RNF183 promoted cell proliferation and conferred resistance to trametinib. **a** mRNA levels of nuclear factor-kappa B (NF-κB) downstream genes in HCT116 cells transfected with small interfering RNAs (siRNAs) of RNF183 (siRNF183) or non-specific control (siNC) were detected by qPCR. IL-8 mRNA level is down-regulated in HCT116 cells transfected with siRNF183. **b** IL-8 mRNA levels are decreased in HT29 and HCT116 cells treated with 20 nmol/L trametinib for 48 h. **c** IL-8 protein levels are down-regulated in HT29 and HCT116 cells treated with 20 nmol/L trametinib for 48 h. Effects of trametinib treatment in combination with RNF183 overexpression or knockdown on IL-8 mRNA (**d**) and protein (**e**) levels in HT29 cells. **f** Effects of RNF183 overexpression with or without IL-8 knockdown on HT29 cell colony formation. **g** HT29 and HCT116 cells were transfected with siNC or IL-8-targeted siRNA (siIL-8) for 24 h; then the cells were treated with 20 nmol/L trametinib for an additional 72 h, and the cell viability was measured with MTT assay. All experiments were repeated at least three times, and data are presented as mean ± SD. *SELE* selectin E, *PLAU* plasminogen activator urokinase, *ICAM1* intercellular adhesion molecule 1, *CXCR4* C-X-C motif chemokine receptor 4, *IL*-*6* interleukin-6, *MMP9* matrix metallopeptidase 9. *P < 0.05, **P < 0.01, ***P < 0.001

It was reported that RNF183 activated the NF-κB pathway in inflammatory bowel disease [16], so we investigated whether downstream target genes of the NF-κB pathway were required for the function of RNF183. We knocked down *RNF183* using siRNA in HCT116 cells and detected the expression of *SELE*, *PLAU*, *ICAM*-*1*, *CXCR4*, *IL*-*6*, *MMP*-*9*, and *IL*-*8* with qPCR. The results indicated that RNF183 silencing significantly decreased the mRNA abundance of IL-8 (*P* < 0.001) (Fig. 3a). Next, we investigated the effects of trametinib on IL-8 expression in HT29 and HCT116 cells with qPCR and ELISA. Results showed that trametinib treatment significantly repressed the expression of IL-8 at both mRNA (Fig. 3b) and protein (Fig. 3c) levels (both *P* < 0.001), and this phenomenon was in accordance with a previous report [25].

Subsequently, we evaluated whether RNF183 affects trametinib-induced IL-8 down-regulation in HCT116 cells. RNF183 overexpression significantly induced *IL*-*8* expression and partly counteracted trametinib-induced repression of *IL*-*8* expression (*P* < 0.05) (Fig. 3d, left). Transfection of RNF183 siRNA significantly repressed IL-8 expression, and administration of trametinib synthetically decreased the abundance of IL-8 mRNA (*P* < 0.001) (Fig. 3d, right). Detection of IL-8 protein concentration in the medium showed similar results (*P* < 0.05) (Fig. 3e).

Because RNF183 expression was induced by trametinib and suppressed trametinib-induced inhibition on cell proliferation, and because IL-8 is a downstream target of RNF183, we evaluated the role of IL-8 in RNF183-enhanced growth of HCT116 cells using colony formation assay. RNF183 overexpression significantly increased the number of colonies, whereas this effect was largely diminished by IL-8 knockdown (*P* < 0.001) (Fig. 3f). Moreover, knockdown of IL-8 synergistically repressed the proliferation of HT29 and HCT116 cells with trametinib treatment (*P* < 0.01) (Fig. 3g). These results suggested that IL-8 was a potential mediator of RNF183 in overcoming trametinib-induced growth inhibition of CRC cells.

### RNF183 conferred resistance to trametinib in vivo

**Fig. 4:**
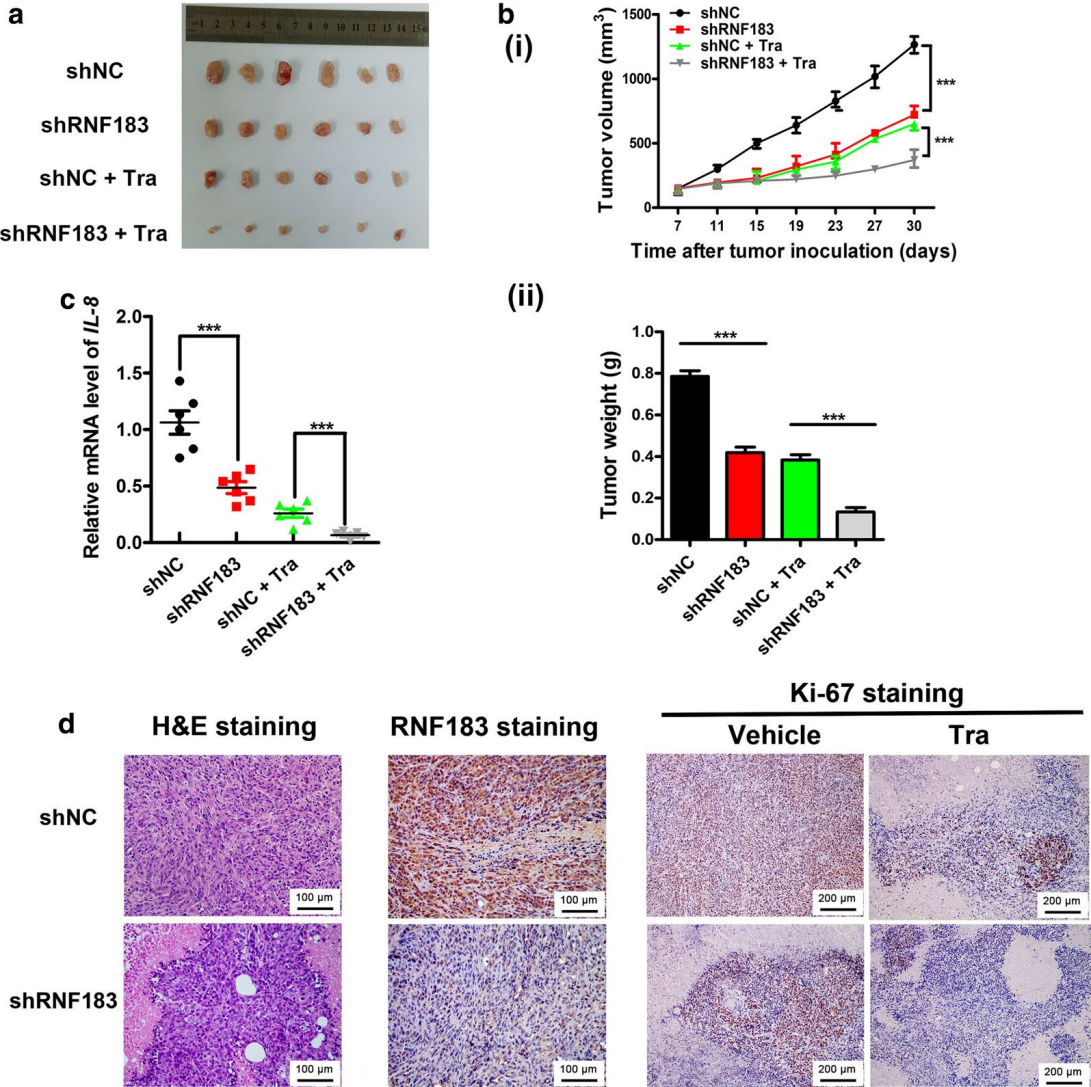
Synergistic antitumor effects of RNF183 knockdown and trametinib treatment on xenograft tumor growth of HCT116 cells. **a** A representative image of tumors collected from mice inoculated with HCT116 cells that were stably transfected with shRNF183 or shNC and treated with or without trametinib. When tumor reached 100 mm^3^, 6 mice respectively from the shRNF183 or shNC groups were given 300 μg/kg trametinib every day for 23 days. The remaining 6 mice in each group were treated with vehicle as control. **b** RNF183 knockdown and trametinib synergistically reduced tumor volumes (**i**) and tumor weights (**ii**). **c** qPCR assay results show that the *IL*-*8* levels in xenograft tumors were synergistically repressed by RNF183 knockdown and trametinib treatment. **d** Images exhibiting the hematoxylin–eosin (H&E), RNF183, and Ki-67 staining for xenograft tumors, which indicate decreased intensity of Ki-67 in RNF183-knockdown, trametinib treatment, and combination treatment groups. RNF183 is located in the cytoplasm and nucleus and Ki-67 is located in the nucleus. ***P < 0.001

Finally, we evaluated the function of *RNF183* in vivo. We injected shNC control and stable RNF183-knockdown HCT116 cells into BALB/c nude mice to establish xenograft models. Then, the mice were subjected to vehicle or trametinib treatment. Results showed that both trametinib treatment and RNF183 knockdown inhibited tumor growth, and their combination showed significant synergistic effects that resulted in the smallest tumor volume (Fig. 4a, b(i)) and lowest tumor weight (Fig. 4b(ii)) (*P* < 0.001). IL-8 levels in these xenograft tumors were monitored using qPCR, and results also showed synthetic effects of shRNF183 and trametinib on repressing *IL*-*8* transcription (*P* < 0.001) (Fig. 4c). Moreover, combined RNF183 knockdown and trametinib treatment markedly decreased the Ki-67 level in xenograft tumors (Fig. 4d), which indicated decreased cell proliferation. These results suggest that RNF183 conferred resistance to trametinib-induced growth inhibition in vivo and IL-8 was a potential downstream target of RNF183 in vivo.

## Discussion

In the present study, we conducted synthetic lethal shRNA screening and showed that RNF183 conferred resistance to trametinib in CRC cells. Moreover, IL-8 was induced by RNF183, and IL-8 knockdown showed synergistic effects with trametinib on inhibiting proliferation of CRC cells.

It has been reported that *BRAF* (B-Raf proto-oncogene) and *KRAS* (KRAS proto-oncogene) mutations in CRC predicted resistance to anti-EGFR (epidermal growth factor receptor) therapy [26], and CRC cells were less responsive to anti-BRAF and anti-MEK therapies [27–29]. The combination of other targeted therapies significantly improved the effects of trametinib. For example, the combination of trametinib and CDK4/6 (cyclin dependent kinase 4/6) inhibitor was shown to be highly efficacious in inhibiting the growth of *KRAS*-mutant CRC cells and inducing the tumor regression in patient-derived xenograft models of CRC [22]. *CDK6* as well as other genes reported to cause trametinib resistance, such as *HDAC* [23] and *FGFR* [24], were also identified in our primary screening. In addition, we found some new genes not previously reported to confer trametinib resistance, including *RNF183*. RNF183 could activate the NF-κB signaling pathway by increasing the ubiquitination and degradation of NF-κB inhibitor α (IκBα), and IL-8 is a downstream target of this proinflammatory signaling pathway [16]. The MAPK signaling pathway has been reported to be involved in regulating *IL*-*8* expression [25]. We found that trametinib treatment significantly decreased IL-8 levels while up-regulating RNF183 expression in both *KRAS*-mutated (HCT116) and *BRAF*-mutated (HT29) CRC cells. Moreover, since IL-8 can be targeted by specific agents such as siRNA and antibody, its role in growth and trametinib resistance of CRC cells suggests that it could serve as a therapeutic target. We further found that, compared with trametinib monotherapy, the combination of RNF183 knockdown with trametinib markedly attenuated the proliferation and increased apoptosis and death of CRC cells, suggesting a synergism of these two treatments. In addition, as a member of E3 ubiquitin ligase containing ring finger domain, RNF183 has been shown to be involved in partial resistance to trametinib in CRC cells. It has been reported that the cancer therapy of inhibition ubiquitin ligase was exciting [30]. Therefore, RNF183 can be a new candidate for further research on ubiquitin ligase and may be a potential target gene for CRC therapy.

There are several limitations in the present study. The coverage of the shRNA library used in our study was not large enough. The library targets 15,000 genes, which covers about a half of human genes. Generation of an shRNA library with more reliable targeting sequences and higher genomic coverage could more accurately identify genes that confer drug resistance. Further research using stable RNF183-overexpressing cells to investigate trametinib resistance in vivo is needed. Furthermore, investigating the role of other candidate genes in trametinib resistance is also required.

## Conclusion

Taken together, our data suggest that RNF183 and IL-8 are potential synergistic targets for anti-MEK therapy for CRC.

## Additional file

**Additional file 1: Table S1.** Abundance of genomic short hairpin RNA (shRNA) fragments under different conditions.
